# Soil accumulation and plant uptake of pharmaceutical active compounds and related metabolites from irrigation water in fennel (*Foeniculum vulgare* Mill.)

**DOI:** 10.3389/fpls.2026.1664441

**Published:** 2026-02-23

**Authors:** Giuseppe Gatta, Francesco De Mastro, Federica Carucci, Michele Perniola, Michele Denora, Gennaro Brunetti, Anna Gagliardi, Marcella M. Giuliani

**Affiliations:** 1 Department of Agricultural Sciences, Food, Natural Resources and Engineering (DAFNE), University of Foggia, Foggia, Italy; 2 Department of Soil, Plant, and Food Science, University of Bari, Bari, Italy; 3 Department of Agricultural and Forestry scieNcEs (DAFNE), University of Tuscia, Viterbo, Italy; 4 Department of Agriculture, Forest, Food and Environmental Sciences, University of Basilicata, Potenza, Italy

**Keywords:** analysis of means, bioconcentration factors, emerging contaminants, hierarchical clustering, wastewater reuse

## Abstract

**Introduction:**

The reuse of treated wastewater (TWW) in agriculture is attracting increasing interest as a sustainable strategy to address water scarcity, particularly in arid and semi-arid regions. However, its use can pose risks due to the potential presence of emerging contaminants of concern, such as personal care products and pharmaceuticals.

**Methods:**

This study investigated the fate of three commonly occurring pharmaceutical contaminants (PhACs) (carbamazepine, climbazole, and flecainide) and their metabolites in the soil–plant system when applied through treated wastewater. The research involved irrigating a fennel crop (*Foeniculum vulgare* Mill.) with fresh water spiked with these PhACs at different concentrations (0.5, 2.0, 200, and 600 µg L⁻¹). Fennel plants were grown under controlled greenhouse conditions and analysed for PhAC content in their roots, leaves, and edible parts (bulbs). Soil and plant PhACs content were evaluated using SPE-UHPLC-HRMS/MS and the Bioconcentration (BCF) and translocation factors (TF) were also assessed.

**Results:**

Results showed PhACs accumulation in the soil and roots only at higher spiked concentrations (≥200 µg L⁻¹). Among the compounds, carbamazepine exhibited the highest root accumulation (BCF>1), but limited translocation to bulbs (TF<1). Climbazole and flecainide, despite their persistence in soil, showed low root uptake (BCF<1) and negligible translocation to bulbs.

**Discussion:**

Multivariate statistical analyses revealed compound-specific patterns governed by physicochemical properties such as ionization and hydrophobicity. Overall, fennel crop showed a restricted capacity to accumulate and translocate PhACs to bulbs, suggesting a physiological barrier that may reduce human health risks when using treated wastewater for irrigation. The results provide new insights into the environmental safety of wastewater reuse, with a specific focus on its impact on crop yield, highlighting the need for crop-specific assessments.

## 1 Introduction

Global water scarcity is intensifying due to climate change, increasing demand, and inadequate management, with the Mediterranean Basin emerging as a major hotspot (Eekhout et al., 2024; Tappi et al., 2023a, 2023). As agriculture accounts for nearly 70% of global freshwater use and 1.8 billion people are expected to face limited water access by 2025, innovative and integrated water management strategies are urgently needed to safeguard food security (Hashem and Qi, 2021; Lahlou et al., 2021; Hamdan et al., 2022; Carucci et al., 2023a).

In this context, the reuse of treated wastewater for irrigation is emerging as an increasingly explored and adopted agronomic strategy, especially in areas facing water scarcity, expanding urban populations, and increasing demand for irrigation water (Winpenny et al., 2010).

Supported by EU Directive 2020/741 (Official Journal of the European Union, 2020), TWW reuse promotes sustainable, climate-resilient farming. Globally, over 20 million hectares are already irrigated with TWW or untreated wastewater, a practice set to grow (Jiménez, 2008; Seidi et al., 2020). Beyond conserving freshwater resources, TWW reuse can enhance soil fertility and crop yields, reduce dependence on chemical fertilizers, and contribute to lower greenhouse gas emissions (Abou Jaoude et al., 2025; Gatta et al., 2015). Despite the steady increase in TWW production and its recognized benefits, its reuse in agriculture remains limited at the global scale. A key barrier to wider TWW use is the presence of unregulated contaminants like pharmaceutical residues and metabolites, which conventional treatment often fails to remove. Antibiotics, antifungals, and antidepressants are especially concerning due to their persistence. Repeated TWW irrigation can lead to their accumulation in soils and potential uptake by edible plants (Mordechay et al., 2022; Ponce-Robles et al., 2022).

Therefore, the greatest risk associated with pharmaceutical active compounds (PhACs) contamination may occur in regions where vegetables are intensively cultivated and irrigated with reclaimed wastewater.

This concern is supported by findings from Tadić et al. (2021), who detected ten antibiotics and their metabolites in lettuce samples collected from fields irrigated with TWW. Similarly, Mordechay et al. (2021) reported the highest number and concentrations of PhACs in leafy vegetables. Specifically, analysis of samples collected from commercial fields irrigated with reclaimed wastewater in Israel revealed that antiepileptics-namely carbamazepine, lamotrigine, and gabapentin-were the most dominant therapeutic group detected across the reclaimed wastewater-soil-plant continuum. These results highlight the importance of understanding the mechanisms underlying the uptake and translocation of pharmaceuticals in plants, usually governed by the physicochemical properties of the compounds (Carter et al., 2014), as well as environmental factors such as soil temperature, pH, and plant-specific parameters like transpiration rates (García et al., 2019).

PhACs uptake depends on factors like biodegradability, which influences soil concentration (Dalkmann et al., 2014; Grossberger et al., 2014), and chemical properties such as lipophilicity and charge (Miller et al., 2016; Li et al., 2019). Moderately lipophilic compounds (octanol/water distribution coefficients, log D 1.5–3) are more likely to move from roots to bulbs, leading to higher concentrations in leaves and fruits (Riemenschneider et al., 2016; Madikizela et al., 2018). Therefore, assessing the fate of PhACs within the plant-soil system is essential, particularly in arid and semi-arid regions of the Mediterranean, where the use of recycled wastewater for irrigation is expected to become increasingly common. This practice may offer a sustainable alternative water source, particularly for high water-demand crops such as fennel. Research on bulbous crops is limited, and to date, fennel has not been studied. The assessment of the uptake and translocation of pharmaceutical active compounds (PhACs) in this crop could be particularly interesting, as it exhibits traits of both root and leafy vegetables. Experimental evidence indicates that pharmaceutical accumulation in crops generally follows the order: fruiting vegetables < cereals and forages < root vegetables < leafy greens (Christou et al., 2019), placing fennel in an intermediate position in terms of potential PhAC accumulation. Fennel (*Foeniculum vulgare* Mill.), a member of the Apiaceae family, is one of the oldest cultivated herbs in the Mediterranean basin. It is a valuable edible, medicinal, and cosmetic plant predominantly grown in arid and semi-arid environments, where water availability is a key factor limiting growth and yield (Bahmani et al., 2015).

Although previous studies have demonstrated that fennel possesses a notable biosorption capacity for certain contaminants, including heavy metals such as cadmium (Cd), nickel (Ni), and chromium (Cr) (Endalamaw and Chandravanshi, 2015; Rao et al., 2010), to the best of our knowledge, this is the first study into the uptake of PhACs by fennel plants irrigated with contaminated water.

In this context, several studies have evaluated the uptake and accumulation of pharmaceutical contaminants in agricultural crops irrigated with treated wastewater, consistently highlighting strong crop- and compound-specific variability (Wu et al., 2015; Christou et al., 2019; Sleight et al., 2023). These studies consistently report a lack of data for aromatic and medicinal crops, including fennel, and very limited information for compounds such as climbazole and flecainide. Consequently, substantial knowledge gaps persist regarding the behavior, uptake, and translocation of these pharmaceuticals in soil–plant systems involving Mediterranean horticultural species such as fennel.

Gaining insight into the accumulation of PhACs in soil and plant tissues is critical not only for environmental risk assessment but also for evaluating their potential impact on irrigated horticultural crops and informing remediation strategies.

Accordingly, the main objective of this study was to address these knowledge gaps by: (i) investigating the fate and behavior of three widely used PhACs as carbamazepine, climbazole, and flecainide and their metabolites within the soil–plant system under fennel cultivation, and (ii) assessing the plant’s capacity to uptake and translocate PhACs.

## 2 Materials and methods

### 2.1 Experimental set-up and crop growth condition

The trial was carried out at the Department of Agricultural Sciences, Food, Natural Resources and Engineering of the University of Foggia (Southern Italy) in a glasshouse. During the experiment period, greenhouse air temperatures ranged from 12–20°C in October to 23–37°C in August.

Single plants of fennel (*Foeniculum vulgare* Mill.) cv ‘Michelangelo’ were cropped into cylindrical polyethylene pots (0.4 m diameter × 0.4 m high) filled with 12 kg of sandy clay loam soil (**Table 1**). Fennel plants were transplanted at the early vegetative stage (3–4 true leaves, with a height of 10–15 cm) into pots and grown throughout the entire experimental period under greenhouse conditions. Plant growth and exposure to pharmaceutical active compounds occurred simultaneously over the full crop cycle.

**Table 1:** Main physico-chemical soil characteristics used for experimental trial.

Parameter	Methods	Values	Units
Physical parameters			
Sand	DIN ISO 11277, 2002	54.1 ± 1.8	% dry weight
Silt	DIN ISO 11277, 2002	26.0 ± 0.8	% dry weight
Clay	DIN ISO 11277, 2002	19.9 ± 1.6	% dry weight
Soil Texture	USDA texture classification	Sandy Loam	–
Field Water Capacity	Plate apparatus (- 0.03 MPa)	34.1 ± 1.9	% dry weight
Wilting Point	Plate apparatus (- 1.5 MPa)	18.3 ± 0.9	% dry weight
Chemical parameters			
pH	1:2.5 (w/v) aqueous soil extracts	8.6 ± 0.06	–
Soil electrical conductivity	1:2 (w/v) aqueous soil extracts	0.45 ± 0.04	dS m^-1^
Organic matter	Walkley and Black (Walkley, 1947)	2.6 ± 0.10	% dry weight
Total carbon content, C	Elemental analysis: ISO 10694, 2021	3.58 ± 0.15	% dry weight
Total nitrogen content, N	Elemental analysis: ISO 13878, 1998	0.16 ± 0.06	% dry weight
Carbon-to-Nitrogen ratio	-	21:1	C:N
Available phosphorus as P_2_O_5_	Olsen method (Olsen, 1954)	75.2 ± 1.6	mg kg^-1^
Total potassium	Coupled plasma optical emission spectrometer. Agilent. ICP-OES 720	16.2 ± 0.5	g kg^-1^
N-NO^3-^	Keeney and Nelson (1982) procedure	6.8 ± 0.3	mg kg^-1^
N-NH_4_^+^	Keeney and Nelson (1982)	10.3 ± 0.4	mg kg^-1^

At the bottom of each pot, a pipe serving as a drainage outlet was used to connect a tank to a drainage reservoir.

Irrigation volumes were estimated based on plant water consumption, which was determined by weighing three representative pots every two days. These pots were selected to reflect different evapotranspiration demands due to variations in incident solar radiation.

The average water consumption was calculated, and irrigation volumes were adjusted to restore soil moisture close to field capacity (**Table 1**) based on gravimetric measurements. Irrigation was applied every three days. Water was supplied manually via surface irrigation, pouring the spiked solution directly onto the soil at the base of each plant, avoiding contact with the leaves to prevent foliar uptake.

The experimental period extended from 26 April to 11 November 2024. This included an initial nursery phase of 45 days, during which fennel seeds were grown to produce seedlings at the 3–4 true-leaf stage for crop transplant. Transplanting into the experimental pots was carried out on 11 June 2024. The fennel growth period under experimental conditions lasted 145 days (145 days after transplanting). Irrigation with spiked water started from transplanting the fennel plants into the pots.

Transplanting was carried out in 2024 on April 26. Fertilization was performed using 2 g pot^-1^ of ternary fertilizer (20-20-20) every 15 days for a total of 26 g pot^-1^. The harvest was carried out at a single time on 11 November 2024.

### 2.2 Irrigation treatment and PhACs concentration adopted

The pharmaceutical active compounds (PhACs) utilized in this study include carbamazepine (CAS number: 298-46-4), climbazole (CAS number: 38083-17-9), and flecainide (CAS number: 54143-55-4). These compounds were chosen due to their frequent presence in treated wastewater (Gatta et al., 2025), their low removal efficiency during conventional wastewater treatment, and to assess the possible formation of their metabolites.

Their persistence raises significant concerns about their presence in treated effluents used for irrigation, particularly regarding potential accumulation in crops and subsequent entry into the food chain. In **Table 2** the main physicochemical parameters of the selected PhACs and metabolites are reported.

**Table 2:** Physicochemical properties (Molecular weight; Water Solubility; log K_OW_, Octanol/Water Coefficient; pKa, Acid Ionization Constant; DT_50_, half-life) of the Selected Pharmaceuticals (PhACs) and metabolites (https://www.chemspider.com).

PhACs	Molecular weight (g mol ^-1^)	Chemical Class	Water Solubility (mg L^-1^) at 25 °C	Log K_OW_	pKa	DT_50_ (day)
**Carbamazepine**	236.2 ^a^	**antiepileptics**	17.6 ^a^	2.45 ^a^	13.9 ^a^	355–1624 ^i^
Acridine	179.22 ^a^	metabolite	slightly soluble in hot water ^a^	3.4^c^	5.6^a^	–
3-Hydroxycarbamazepine	252.27 ^b^	metabolite	non-aq.^b^	2.41 ^b^	9.19 ^b^	–
10,11-dihydro-10-hydroxycarbamazepine	254.28 ^b^	metabolite	non-aq.^b^	0.93 ^b^	12.8 ^b^	–
Carbamazepine 10,11-epoxide	252.27 ^b^	metabolite	non-aq.^b^	1.0 ^b^	16 ^b^	–
10,11-dihydro-10,11-dihydroxy carbamazepine	270.10^d^	metabolite	non-aq.^b^	0.81 ^d^	12.7^e^	–
**Climbazole**	292.7 ^a^	**antifungal**	8.2^f^	3.76 ^a^	6.5^g^	120
OH-Climbazole	294.8 ^h^	metabolite	non-aq. ^h^	3.5 ^h^	–	–
**Flecainide**	414.3 ^a^	**antiarrhythmic**	1.0 ^a^	3.78 ^a^	9.3 ^a^	360

^a^https://pubchem.ncbi.nlm.nih.gov; ^b^Malvar et al., 2020; ^c^Sauvêtre et al., 2018; ^d^Fenet et al., 2012; ^e^Brieudes et al., 2016.

^f^https://sitem.herts.ac.uk/aeru/iupac/Reports/2454.htm?utm.

^g^https://go.drugbank.com/drugs/DB15580?utm.

^h^https://www.ebi.ac.uk/chembl.

^i^Gworek et al., 2021.

Primary compounds of the PhACs are shown in bold.

The experimental design focused on comparing four concentration levels (0.5, 2.0, 200 and 600 μg L^-1^) for each PhACs under study (**Table 3**). Each PhAC was applied individually, and no mixtures were used.

**Table 3:** PhACs (carbamazepine, climbazole, and flecainide) concentration applied by crop irrigation.

Irrigation treatment	Acronymous	Spiked PhAC concentration ^†^ (μg L^-1^)	Irrigation volume applied (L pot^-1^)	PhACs applied by irrigation (μg pot^-1^)
Freshwater	Control_0.0_	–	9.8	–
Freshwater + [0.5 μg L^-1^] PhACs	Low_0.5_	0.5	9.8	4.9
Freshwater + [2.0 μg L^-1^] PhACs	Low_2.0_	2.0	9.8	19.6
Freshwater + [200.0 μg L^-1^] PhACs	High_200_	200	9.8	1960
Freshwater water + [600.0 μg L^-1^] PhACs	High_600_	600	9.8	5880

^†^Concentrations are relative to each PhAC considered (carbamazepine, climbazole, and flecainide).

Freshwater (FW) had been spiked with target contaminants at low doses of 0.5 and 2.0 μg L^-1^, which are comparable to the average concentrations detected in previous field experiments (Gatta et al., 2025). Additionally, FW was enriched with PhACs at high concentrations (200 and 600 μg L^-^¹), deliberately exceeding typical levels detected in wastewater. These elevated doses were applied to simulate soil accumulation resulting from repeated long-term irrigation with pharmaceutical-contaminated water, thereby enabling the investigation of soil–plant interactions under excessive stress conditions and a clearer elucidation of the transformation processes of the parent compounds (Denora et al., 2023, 2024).

The spiking procedure for PhACs was carried out only with the primary forms (i.e., carbamazepine, climbazole and flecainide).

The determination of PhACs and their metabolites was performed on the different matrices (soil and plant) after the irrigation water supply. The standard solution (1000 ppm) was prepared using components with a purity of over 98%, provided by Lab Instruments (Castellana Grotte, Italy). This solution was used to spike FW at both low and high concentrations. The different irrigation treatments were compared with a control group that received only uncontaminated fresh water. The experimental irrigation water treatments were arranged using a randomized block design, with three repetitions.

### 2.3 Sampling and analysis of soil and plant PhACs content

Before the experimental trial started, soil samples were collected from all plots to assess the possible initial soil PhACs and related metabolites content. Moreover, soil samples were taken at the end of the crop cycle. These samples were air-dried, passed through a 2 mm sieve, and stored at –20 °C until laboratory analysis.

PhACs and related metabolites were extracted from soil, as described by De Mastro et al. (2022) and fennel plants using modified QuEChERS method.

The roots were washed with fresh water, rinsed with deionized water, and gently dried before storage. Plant tissues (roots, leaves, and bulb) were chopped and stored in 50 mL centrifuge tubes at –20°C until extraction.

Samples (2 g of roots and leaves, and bulb) were hydrated with 6 mL of water, vortexed for 1 minute, and extracted with 10 mL of acetonitrile. After shaking, a salting-out step was performed using a citrate buffer (4 g MgSO_4_, 1 g NaCl, 0.5 g sodium citrate sesquihydrate, 1 g sodium citrate dihydrate), followed by manual shaking for 5 minutes and centrifugation (5 min at 3700 rpm). The supernatant (6 mL of the ACN layer) was cleaned using 900 mg MgSO_4_ + 150 mg PSA (for roots) or 900 mg MgSO_4_ + 150 mg PSA + 150 mg C18 (for leaves and marketable bulb). After vortexing and centrifugation (5 min at 4000 rpm), the extracts were filtered (PVDF, 0.22 µm), and 1.5 mL aliquots were transferred to vials for analysis.

Quantification of PhACs in the extracts and water samples was carried out using a UHPLC system (Ultimate 3000, Thermo Fisher Scientific) coupled with a TripleTOF 5600+ mass spectrometer (AB Sciex), equipped with a DuoSpray™ ion source operated in electrospray ionization mode. Full details of the LC-MS/MS conditions are reported in De Mastro et al. (2023).

Limits of quantification (LOQ) as well as detection (LOD) and absolute recovery percentage for soil and plants are listed in **Supplementary Tables S1**.

### 2.4 Bioconcentration and translocation factor evaluation

The bioconcentration factor (BCF) is an index that reflects the accumulation of specific PhACs in plant tissues with respect to their concentration in the soil. It was calculated according to the method described by Camacho-Arévalo et al. (2021), as shown in Equation 1:

(1) $$\text{BCF}={\text{C}}_{\text{plant}}/{\text{C}}_{\text{soil}}$$

where C_plant_ and C_soil_ represent the concentrations (ng/g) of particular PhACs in the fennel parts (root, leaf and bulb) and the soil, respectively, on a dry weight basis.

The translocation factor (TF) indicates the extent to which PhACs are translocated from the roots to other parts of the plant. It is calculated by comparing the concentrations (ng/g) of PhACs in the aerial parts (leaf and bulb) to their concentration in the roots, as defined in Equation 2 (Mininni et al., 2024; Camacho-Arévalo et al., 2021):

(2) $$\text{TF}={\text{C}}_{\text{leaf}/\text{bulb}}/{\text{C}}_{\text{root}}$$

These indices are critical for understanding the environmental fate of PhACs in agroecosystems. The BCF provides insight into the plant’s ability to uptake contaminants from the soil, while the TF (performed on a dry weight basis) helps assess the potential for contaminant mobility within the plant and their eventual entry into the food chain. High values of these factors may indicate a greater risk of human exposure through crop consumption or transfer through trophic levels.

### 2.5 Statistical analysis

The dataset was evaluated for compliance with ANOVA assumptions using the Shapiro-Wilk test for data normal distribution and Bartlett’s test for homogeneity of variance. The concentrations of PhACs in both plant and soil, as well as the bioconcentration factor (BCF) and translocation factor (TF) values, did not meet these assumptions. Therefore, the results are presented as mean ± standard error.

Principal component analysis (PCA) was applied as a multivariate statistical approach to better understand the PhAC concentrations in soil and plant tissues, including irrigation treatments and the sample matrix (soil and plant parts) as qualitative supplementary variables (Mongiano et al., 2018). These qualitative variables did not contribute to the computation of principal components but were projected a posteriori as the centroids of the corresponding qualitative variables. Prior to PCA, all variables were centered and scaled. PCA was performed by diagonalizing the correlation matrix and extracting the associated eigenvalues and eigenvectors.

Hierarchical Clustering on Principal Components (HCPC) was then conducted using Euclidean distance and Ward’s method to identify groups of observations with similar profiles (Carucci et al., 2023b). Analysis of Means (ANOM) was used to verify significant differences (α=0.05) between mean values of the groups for each PhAC considered.

All analyses were performed using JMP software, version 16.2 (SAS Institute Inc., Cary, NC, USA), and graphical representations were generated using SigmaPlot software (Systat Software Inc., Chicago, IL, USA).

## 3 Results

### 3.1 PhACs and metabolites soil-plant concentrations

**Figure 1:**
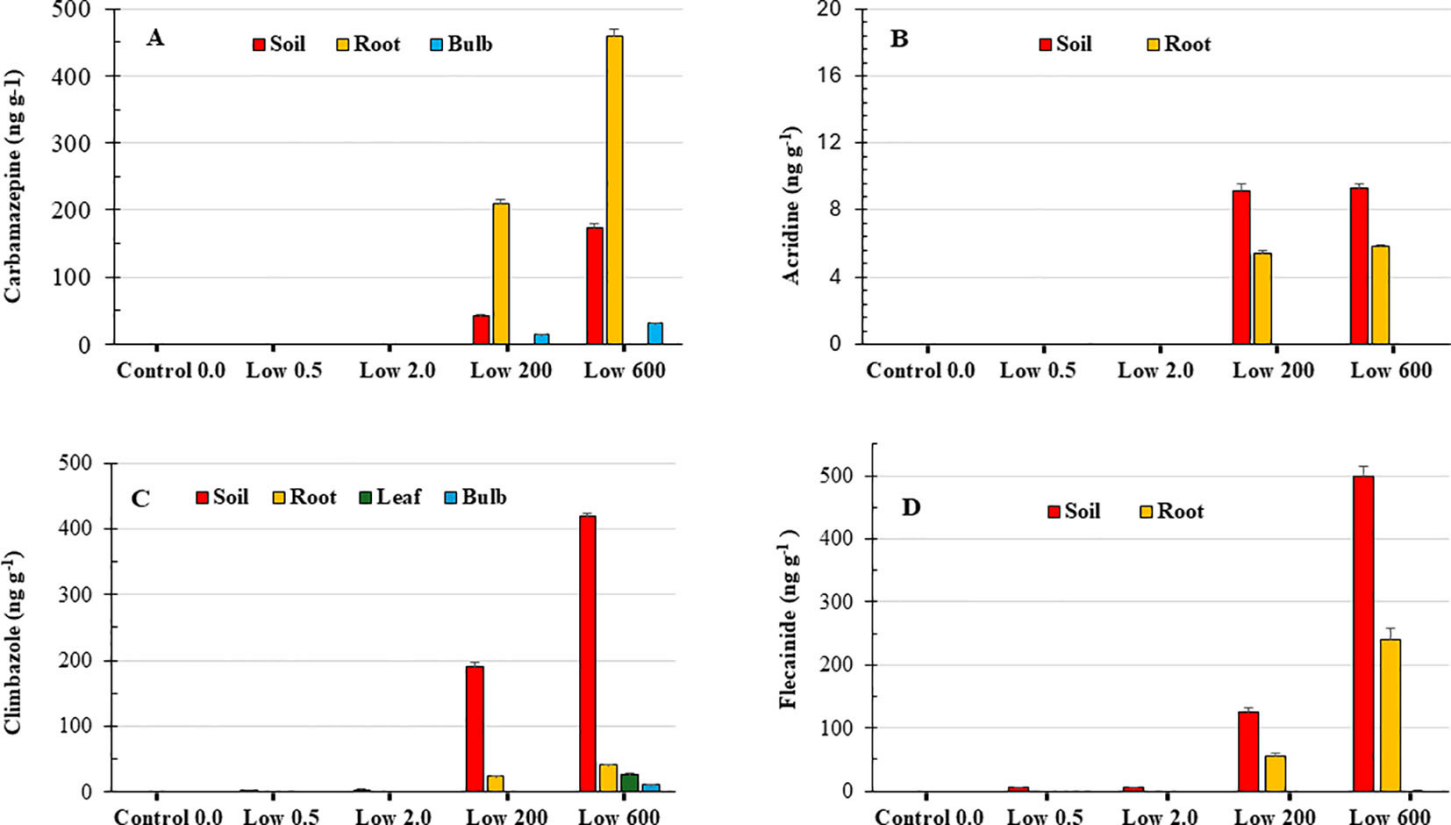
Mean concentrations of carbamazepine **(A)**, acridine **(B)**, climbazole **(C)**, and flecainide **(D)** expressed as ng g^-^¹ dry weight in soil and plant tissues (root, leaf, and bulb) under different irrigation water treatments. Values are reported as mean ± standard error (n = 3). PhAC concentrations for specific treatments or plant tissues not shown in the figure were below the limit of quantification (LOQ).

As expected, all PhACs showed higher concentrations in the soil under the High_200_ and High_600_ treatments compared to the Low_0.5_ and Low_2.0_ treatments. **Figure 1** and **Supplementary Table S2** show the mean values of PhACs and metabolite concentrations detected in the soil and plant (root, leaf and bulb) at the end of the experiment. Among the high-concentration treatments (High_200_ and High_600_), climbazole and flecainide exhibited the most significant accumulation, with concentrations of 418.7 ± 4.3 ng g^-1^ and 497.9 ± 15.9 ng g^-1^ in High_600_, and 190.5 ± 5.6 ng g^-1^ and 124.6 ± 7.4 ng g^-1^ in High_200_, respectively. In contrast, for carbamazepine and its metabolite (acridine), the concentrations in the Low_0.5_ and Low_2.0_ treatments were below the limit of quantification (<LOQ).

The concentration of PhACs varied according to both the plant part and the irrigation treatment applied (Low_0.5_, Low_2.0_, High_200_ and High_600_). For all the compounds examined, with the exception of carbamazepine and climbazole, the content in the bulbs of PhACs was below the detection limit (<LOQ). No traces of the metabolites of carbamazepine, climbazole and flecainide were detected in the soil and in the different parts of the plant.

### 3.2 Multivariate statistical analysis on PhACs soil and plant contents

#### 3.2.1 Principal component analysis

**Figure 2:**
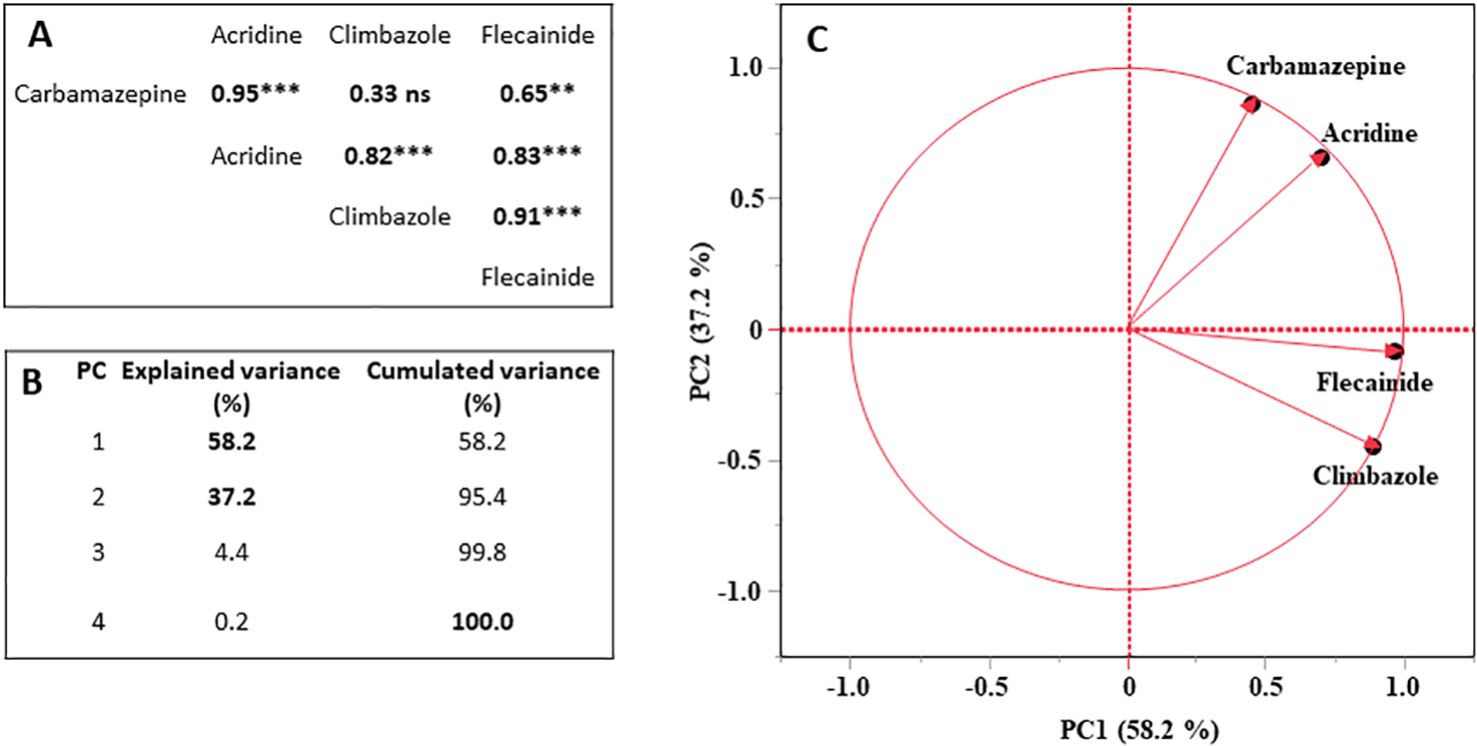
Correlation matrix with Pearson’s r values **(A)**, explained variance **(B)** of the principal component analysis, and biplot of variables **(C)** of PhAC (carbamazepine, acridine, flecainide and climbazole).

The PCA allowed for the evaluation of which PhACs contribute most to the differences between irrigation treatments and plant parts, when considered simultaneously. The correlation matrix and the PCA biplot are reported in **Figure 2**. Pearson correlation coefficients among different PhACs were significant at p ≤ 0.05, except carbamazepine *vs* climbazole comparison. Higher positive correlations (0.95) emerged between carbamazepine and acridine in different matrix considered in PCA analysis (soil and plant) and between climbazole and flecainide (0.91) (**Figure 2A**). The first two principal components accounted for nearly all of the experimental variability, indicating robust dimensional reduction. As highlighted in **Figure 2B**, the first two components explain almost all of the variability in the experimental data (95.4%). Subsequently, the characterization of PCs was performed by calculating correlation coefficients with the quantitative variables and the associated significance level (**Table 4**).

**Table 4:** Pearson’s correlation coefficients between active quantitative variables and the first two Principal Components (PC), with indication of the explained variance.

Variable	Principal component	
	PC_1_	PC_2_
Carbamazepine	0.52 ^**^	0.79 ^***^
Acridine	0.50 ^**^	0.59 ^**^
Climbazole	0.89 ^***^	-0.44 ^*^
Flecainide	0.96 ^***^	-0.014 ^ns^
Explained variance (%)	58.3%	37.2%

Significance codes: ^***,^ significance at *p<*0.001; ^ns^ not significant.

The PCA results identified two factors that explain 58.2% and 37.2% of the total variance, respectively. PC_1_ mainly represented compounds strongly retained by soil, particularly climbazole and flecainide. Indeed, the PCA revealed that the first factor (PC_1_) was strongly and positively correlated with climbazole and flecainide (p-value <0.01). This correlation suggests that PC_1_ could represent a factor linked to the physic-chemical characteristics (with special regard to log K_OW_ values) of climbazole and flecainide can be considered as ‘*soil-absorbed form*’ factor.

On the contrary, the second factor (PC_2_) described compounds with lower soil affinity, dominated by carbamazepine behavior. PC_2_ was positively associated with carbamazepine (p-value <0.01). PC_2_ can be considered a factor linked to the ‘*low soil-absorbed form*’. Finally, the acridine, carbamazepine metabolite compound, resulted positively associated with both factor 1 and 2.

The qualitative variable projection confirmed that soil-absorbed PhACs were associated with high PhAC concentrations in irrigation water, whereas plant tissues were associated with low-absorption compounds.

The barycenter’s positions of the supplementary qualitative variables are reported in **Table 4**. PC_1_ (‘*soil-absorbed form*’ of PhACs) shows higher significant positive correlations with PhAC concentrations in soil, as well as with the High_200_ and High_600_ PhAC levels in irrigation treatment, and significantly and negative correlation with PhACs concentration in leaf and bulbs, and with Control_0.0_, Low_0.5_ and Low_2.0_. PC_2_ (‘*low soil-absorbed form*’ of PhACs) displays a negative and significant correlation with soil and a positive one with roots, while its correlations with the different PhAC concentration levels are not significant.

#### 3.2.2 Hierarchical clustering on principal components

**Figure 3:**
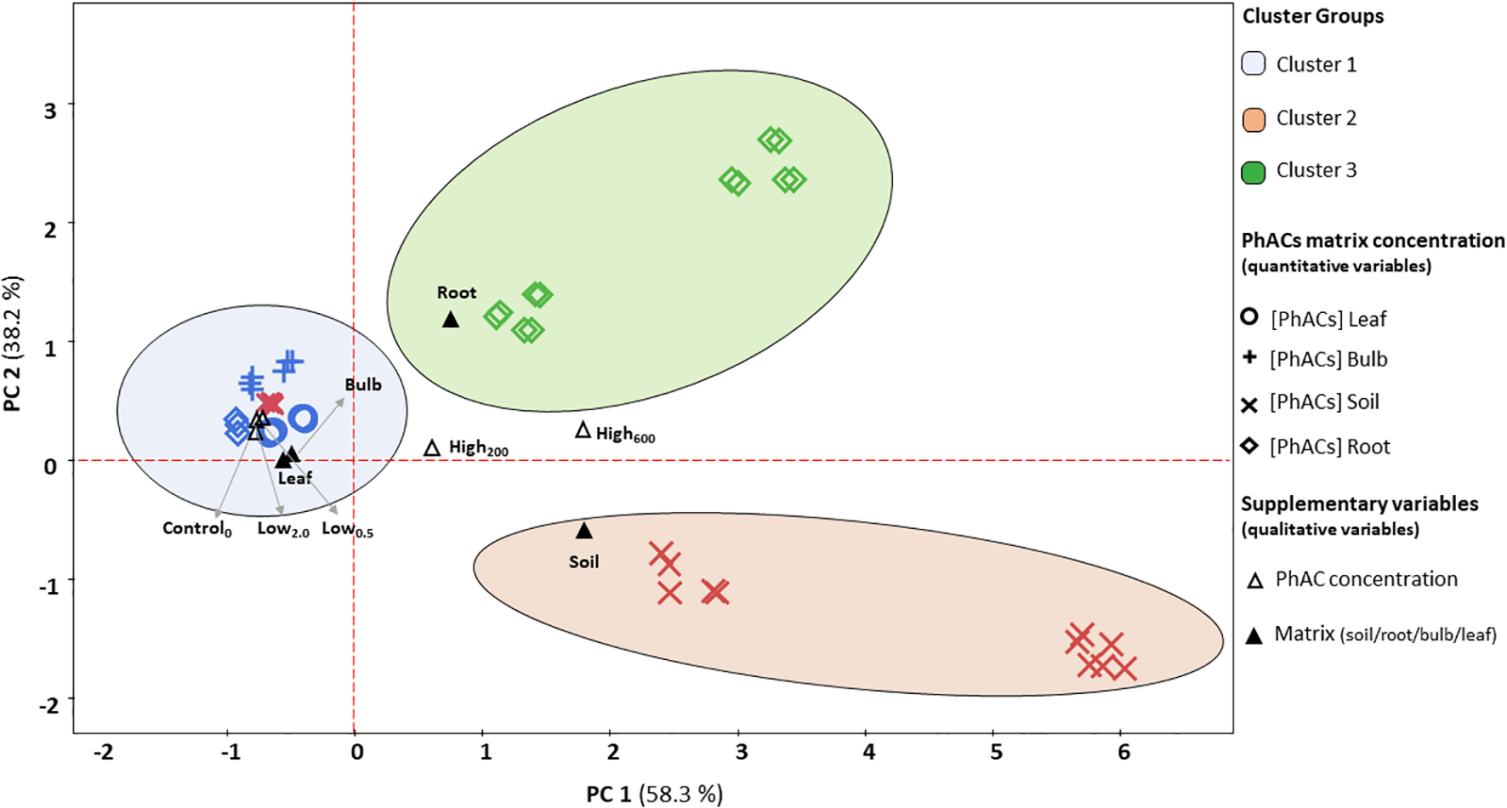
PCA biplot with cluster delimitation based on quantitative variables (PhAC concentrations in soil, root, bulb and leaf) and qualitative supplementary variables (irrigation treatment and matrix). The barycenter’s of the supplementary variables that contribute most to cluster variance are indicated by ▴ for different matrices (leaf, root, bulb, and soil) and by Δ for different concentrations of PhACs (Control_0.0_, Low_0.5_, Low_2.0_, High_200_, and High_600_). The concentrations of the PhAC compounds in the various matrices are represented as follows: ○ for leaves, + for bulb, × for soil, and ◊ for roots. The clusters are painted in different colors and identified using hierarchical clustering with Ward’s method and Euclidean distance.

HCPC clearly distinguished low-exposure plant tissues from high-exposure soil and root samples, forming three well-defined clusters. HCPC performed three clusters on the extracted principal components (**Figure 3**). Cluster 1 contained the highest number of analyzed PhAC samples, with 48 values (**Table 5**), generally corresponding to the control treatment (Control_0.0_), irrigation treatments with low PhAC concentrations (Low_0.5_ and Low_2.0_), and bulb and leaf part of the plants as matrix. In this cluster, the most prevalent PhACs is carbamazepine (3.2 ± 1.3 ng g^-^¹) followed by climbazole (2.7 ± 0.9 ng g^-^¹).

**Table 5:** PhACs content in the different clusters identified following the multivariate HCPC procedure.

PhCA	Cluster	n. sample	Mean value	Confidence interval (95%)	
				Lower limit	Upper limit
Carbamazepine	1	48	3.2 ± 1.3	0.5	5.8
	2	6	108.2 ± 29.3	32.8	183.6
	3	6	333.8 ± 56.1	189.4	478.1
Acridine ^†^	1	48	0.01 ± 0.004	0.0005	0.020
	2	6	9.1 ± 0.11	8.9	9.4
	3	6	5.5 ± 0.13	5.23	5.9
Climbazole	1	48	2.7 ± 0.9	0.7	4.7
	2	6	304.6 ± 51.1	173.1	436.0
	3	6	32.4 ± 4.1	21.9	42.9
Flecainide	1	48	0.6 ± 0.2	0.1	1.2
	2	6	311.3 ± 83.8	95.8	526.8
	3	6	148.2 ± 42.1	39.9	256.5

^†^metabolite of the carbamazepine.

The mean values ± standard error and the upper and lower limits related to the 95% confidence interval are reported.

Cluster 1 was located on the negative side of PC_1_ indicating a weak correlation with the accumulation of climbazole and flecainide in leaf and bulb matrix (**Figure 3**).

Clusters 2 and 3 were both characterized by a low number of PhAC samples, with 6 values each (**Table 5**), corresponding to the treatments with high concentrations (High_200_ and High_600_).

Cluster 2 was positioned in the positive side of PC_1_ and in the negative side of PC_2_ being characterized by high concentrations of climbazole (304.6 ± 51.1 ng g^-^¹) and flecainide (311.3 ± 83.8 ng g^-^¹), particularly in the soil. In contrast, cluster 3 was positioned in the positive side of both PCs including all the PhACs under study, especially carbamazepine (333.8 ± 56.1 ng g^-^¹), located in the root matrix (**Table 5**; **Figure 3**).

An Analysis of Means (ANOM) was conducted to evaluate significant differences among the cluster means identified by HCPC, providing a clearer understanding of the results presented in **Table 5** and **Figure 3**. ANOM confirmed statistically significant differences among cluster means for nearly all compounds.

**Figure 4:**
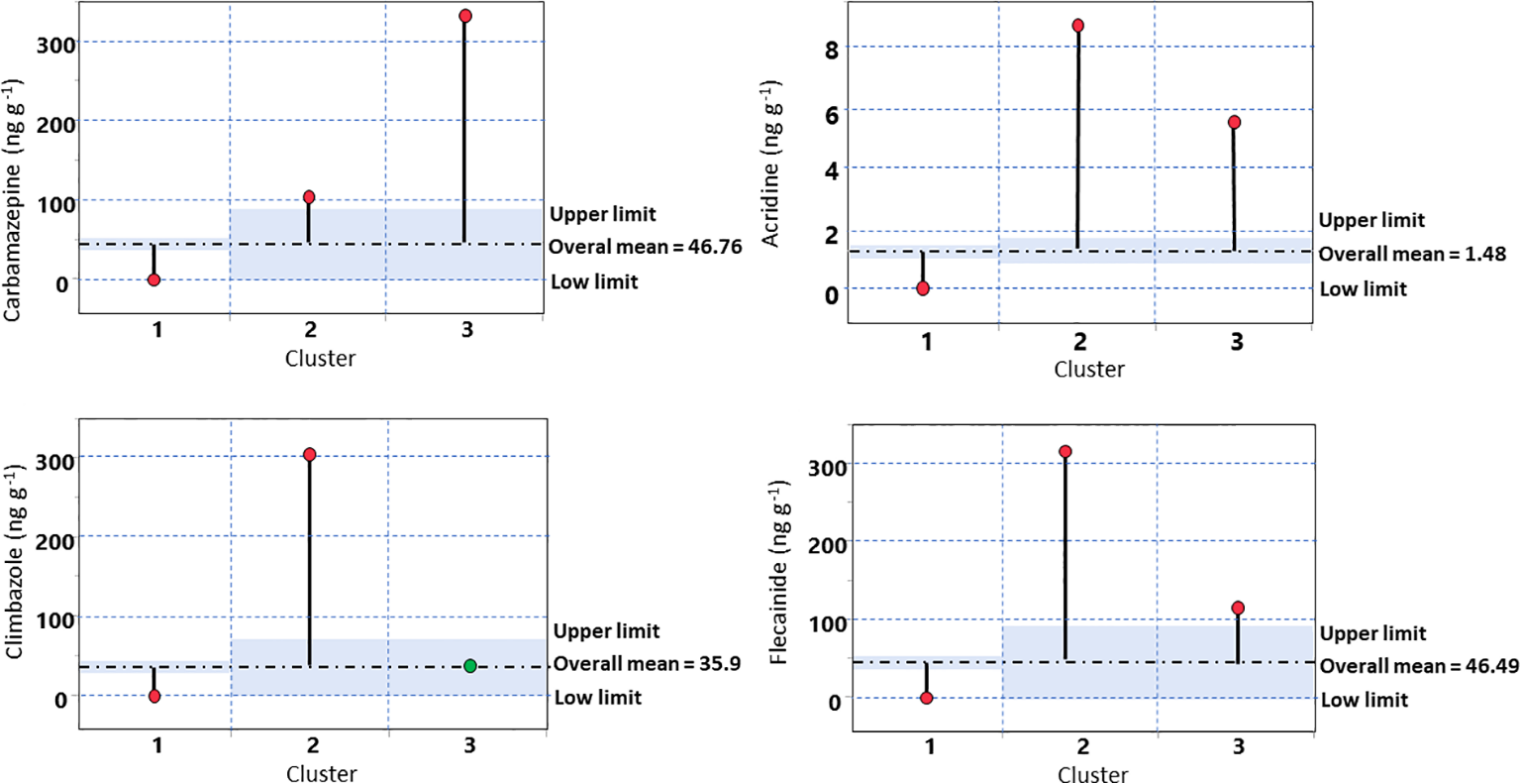
ANOM (Analysis of Means) decision chart showing the comparison of group/cluster means for the different PhACs, against the overall mean. The central line (dotted line) represents the overall mean, while the upper and lower decision limits (α=0.05) indicate thresholds for statistical significance. Group means falling outside these limits differ significantly from the overall mean.

The **Figure 4** presents the ANOM decision chart for the different PhACs analyzed. The mean values among the three clusters were statistically different (α=0.05) for all PhACs, except for climbazole relative to the cluster 3. For all PhACs, cluster 1 showed mean values below the overall mean, while clusters 3 and, especially, cluster 2 displayed mean values above the overall mean.

### 3.3 Bioconcentration and translocation factors

Plant bioconcentration occurred only under high PhACs levels and was mainly restricted to roots. **Table 6** gives the bioconcentration factor (BCF) for the PhACs detected in plant tissues and reports how they are affected by the irrigation treatments (Control_0.0_, Low_0.5_, Low_2.0_, High_200_ and High_600_).

**Table 6:** Bioconcentration factor (BCF) values of pharmaceuticals (PhACs) in different plant parts (root, leaf and bulb) related to different irrigation water treatments irrigation treatments (Control_0.0_, Low_0.5_, Low_2.0_, Low_200_ and Low_600_). For each PhACs, data are the mean ± standard error (3 replications).

Irrigation treatment ^†^	PhACs			
	Carbamazepine	Acridine	Climbazole	Flecainide
Root				
Control_0.0_	0.0 ± 0.0	0.0 ± 0.0	0.0 ± 0.0	0.0 ± 0.0
Low_0.5_	0.0 ± 0.0	0.0 ± 0.0	0.0 ± 0.0	0.0 ± 0.0
Low_2.0_	0.0 ± 0.0	0.0 ± 0.0	0.0 ± 0.0	0.0 ± 0.0
High_200_	4.88 ± 0.23	0.59 ± 0.02	0.12 ± 0.04	0.45 ± 0.015
High_600_	2.66 ± 0.07	0.62 ± 0.01	0.10 ± 0.01	0.48 ± 0.031
Leaf				
Control_0.0_	0.0 ± 0.0	0.0 ± 0.0	0.0 ± 0.0	0.0 ± 0.0
Low_0.5_	0.0 ± 0.0	0.0 ± 0.0	0.0 ± 0.0	0.0 ± 0.0
Low_2.0_	0.0 ± 0.0	0.0 ± 0.0	0.0 ± 0.0	0.0 ± 0.0
High_200_	0.0 ± 0.0	0.0 ± 0.0	0.0 ± 0.0	0.0 ± 0.0
High_600_	0.0 ± 0.0	0.0 ± 0.0	0.07 ± 0.007	0.0 ± 0.0
Bulb				
Control_0.0_	0.0 ± 0.0	0.0 ± 0.0	0.0 ± 0.0	0.0 ± 0.0
Low_0.5_	0.0 ± 0.0	0.0 ± 0.0	0.0 ± 0.0	0.0 ± 0.0
Low_2.0_	0.0 ± 0.0	0.0 ± 0.0	0.0 ± 0.0	0.0 ± 0.0
High_200_	0.32 ± 0.04	0.0 ± 0.0	0.0 ± 0.0	0.0 ± 0.0
High_600_	0.21 ± 0.01	0.0 ± 0.0	0.02 ± 0.001	0.0 ± 0.0

^†^ Control_0.0_, control by Irrigation with fresh water; Low_0.5_, fresh water spiked with PhAC at a concentration of 0.5 µg L^−1^; Low_2.0,_ fresh water spiked with PhACs at a concentration of 2 µg L^−1^; High_200_, fresh water spiked with EC at a concentration of 200 µg L^−1^; High_600_, fresh water spiked with PhACs at a concentration of 600 µg L^−1^.

Under low PhAC concentrations (Low_0.5_ and Low_2.0_), the BCF values were zero for all PhACs and in all matrices considered (root, leaf, and bulb). At higher PhAC concentrations (High_200_ and High_600_), the BCF indicated higher accumulation of carbamazepine in the roots than in the bulb (4.88 ± 0.23 and 2.66 ± 0.07, respectively). For the other PhACs under study, the BCF was higher than 0 but lower than 1 only in the roots.

To assess the translocation potential of the PhACs from roots to aerial plant parts (leaves and bulbs), the translocation factor (TF) was calculated as the ratio between the compound concentration in the aerial tissues and in the roots (**Supplementary Table S3**). The PhACs translocation from roots to aerial tissues was minimal for all investigated compounds. In treatments Control_0.0_, Low_0.5_, and Low_2.0_, PhAC concentrations were below the limit of quantification (**Supplementary Table S2**) in all plant compartments (roots, leaves, and bulbs), and thus TF values could not be determined. TF values exceeding 1 indicate preferential upward mobility of the compound from the roots to aerial tissues (Fernandes et al., 2024).

The results demonstrated a limited translocation capacity of the investigated compounds in fennel plants. All PhACs exhibited TF values well below 1, indicating minimal systemic mobility. Only carbamazepine and climbazole displayed marginally higher TF values (<1) for both leaves and bulbs under the High_200_ and High_600_ treatments, suggesting a modest degree of root-to-bulb transfer.

## 4 Discussion

The recent EU Regulation 2020/741 on treated wastewater reuse for irrigation is based on the estimation and management of environmental impacts with particular reference to the agricultural systems. Therefore, PhACs in treated wastewater and their persistence, bioaccumulation in the soil-plant system, and potential toxicity highlight the need for effective monitoring and management strategies. To the best of our knowledge, no literature is available about the effects of PhACs derived to treated wastewater irrigation on fennel crop. Thus, our study aimed to evaluate the fate of selected pharmaceuticals at different concentrations applied to the fennel crop through irrigation, also considering important factor as BCF and TF. Several studies have reported that a BCF ≤ 1 suggests that, although the plant may uptake pollutants (Ackah et al., 2014; Gatta et al., 2018), including emerging contaminants (Sunyer-Caldú et al., 2022), there is no significant accumulation in plant tissues. Conversely, a BCF>1 indicates that the plant accumulates PhACs in the plant parts. Therefore, low BCF values suggest limited or no uptake of PhACs, implying that the plant tends to exclude these compounds from its tissues.

Under our experimental conditions, all PhACs (carbamazepine, climbazole, and flecainide) were detected in soil following irrigation with spiked fresh water, depending on the different concentrations utilized.

Carbamazepine accumulated in the soil only at High_200_ and High_600_ treatments. In contrast, climbazole and flecainide were detectable in the soil even at lower concentrations (Low_0.5_ and Low_2.0_). Although a complete quantitative mass balance was beyond the scope of this study, a qualitative evaluation suggests that the majority of the applied pharmaceuticals were retained in the soil, particularly for climbazole (about 94.1% and 72.6% for High_200_ and High_600_ treatments, respectively) and flecainide (about 64.8% and 83.4% for High_200_ and High_600_ treatments, respectively), while a smaller fraction was taken up by plant roots (<0.03%) and only negligible amounts were translocated to edible tissues. The mass balance for climbazole confirms its high sorption affinity for the soil matrix and its low mobility (Denora et al., 2023).

Additional fractions may have undergone degradation, irreversible sorption to soil organic matter, or remained unaccounted for due to transformation processes, as previously reported for pharmaceutical contaminants in soil–plant systems (Dalkmann et al., 2014; Meffe et al., 2021; Mordechay et al., 2022).

The soil behavior of flecainide and climbazole may be attributed to similar octanol/water partition coefficient (i.e., log K_OW_) values to those of carbamazepine (3.76 and 3.78 *versus* 2.45). This means that they could interact strongly with the organic component of the soil (Richter et al., 2013), increasing their adsorption in the soil and explaining the high concentration found also in the soil.

These results are confirmed by the multivariate HCPC procedure since climbazole was highly represented in cluster 2 characterized mainly by soil samples. This is a significant result since this compound showed the lowest BCF factor in the roots, highlighting the lowest uptake from the soil to the root. The high flecainide soil content is in accordance with Meffe et al. (2021), who reported a high affinity of flecainide accumulating in the soil. However, our results showed a higher uptake of flecainide from soil to root with respect to climbazole, even if the bioaccumulation coefficient (BCF) was<1 (0.48 and 0.45 for High_600_ and High_200_ irrigation treatment, respectively), thus showing no significant accumulation in this plant part. Flecainide is most represented in cluster 2 characterized mainly by root samples, but the compost seems to remain localized in the root since there was no translocation from the root to the edible bulb (TF_(bulb/root)_=0). These results are relevant since soil-root uptake and plant translocation of pollutants is one of the fundamental processes through which humans are exposed to PhACs through the food chain (Prosser and Sibley, 2014; Fernandes et al., 2024; Petruzzelli et al., 2025).

In our experimental trial, no compounds attributable to the transformation products of flecainide and climbazole were detected in the soil, likely due to different metabolic processes compared to those occurring in humans. The degradation pathways of climbazole and flecainide have primarily been documented in human plasma and urine (Schönrath et al., 2024; Benijts et al., 2003). Climbazole is metabolized through the reduction of its ketone group to a secondary alcohol function, resulting in OH-climbazole (Schmidtkunz et al., 2021), while flecainide undergoes biotransformation via O-dealkylation, producing meta-O-dealkylated flecainide and the corresponding meta-O-dealkylated lactam derivative (Benijts et al., 2003). Furthermore, the lack of metabolites attributable to these two molecules may also be attributable to the duration of the experiment.

The lower concentration of carbamazepine in soil, as compared to climbazole and flecainide, may be attributed to its neutral form and relatively low octanol-water partition coefficient (log K_ow_=2.45), which indicates higher hydrophilicity. A low log K_ow_ value reflects a greater affinity for the aqueous phase, suggesting that carbamazepine is more likely to remain in the soil solution and therefore more inclined to leaching into groundwater (Wojsławski et al., 2019). Due to its higher pKa (13.9) respect to flecainide (9.3) and climbazole (6.49), carbamazepine remains mostly in its neutral (non-ionized) form under typical soil pH conditions, resulting in weaker interactions with soil constituents. For this reason, PCA results showed a high correlation of carbamazepine with PC_2_ representing ‘*low soil-absorbed form*’ factor.

Regarding carbamazepine metabolites, the detection of acridine in soil, even at low concentrations and as the sole metabolite, raises concerns about its potential long-term entry into the food chain and associated toxic effects. Acridine is known to inhibit DNA repair and impair cellular growth. Notably, previous research has shown that acridine is significantly more toxic than carbamazepine across multiple trophic levels (De Mastro et al., 2024).

Among the three compounds, carbamazepine appears to be more readily taken up by roots; indeed, also this compound was highly represented in cluster 3 characterized mainly from root samples. Its concentration in root tissues was approximately 4.8-fold and 2.6-fold higher than respect to soil for the High_200_ and High_600_ irrigation treatments, respectively. BCF was therefore found to be higher for High_200_ than for High_600_. This behavior has also been observed by some authors (Sunyer-Caldú et al., 2022; Fernandes et al., 2022), highlighting how obtaining higher BCF values in experimental treatments with lower contaminant exposure was possible. On the other hand, if a compound’s plant uptake is considered a rate-limited process, BCF values will become progressively smaller as greater contaminant inputs are applied (Fernandes et al., 2024).

Carbamazepine, climbazole, flecainide and related metabolites were not quantified (below the limit of quantification) in the different plant parts (roots, leaves, and bulb) under low-concentration irrigation treatments (**Supplementary Table S2**). Therefore, these compounds’ translocation factor (TF) values were not determined under such treatments (**Supplementary Table S3**). In contrast, under high-concentration irrigation treatments (High_200_ and High_600_), only carbamazepine and climbazole exhibited translocation and accumulation in the aboveground plant parts (leaves and bulb), although the TF values remained well below unity (<1). Since the TF is defined as the ratio of the PhAC concentration in the aerial parts to that in the roots, these results indicate that carbamazepine and climbazole had very low concentrations in the edible bulb compared to the roots, reflecting limited root-to-bulb translocation. These findings are consistent with previous studies (Prosser et al., 2014; Gatta et al., 2025), which reported TF values for aboveground crop parts mostly below 1. For instance, TF values for green pepper, carrot, cucumber, tomato, radish, and lettuce were ≤0.33. These results suggest a physiological barrier that limits PhAC accumulation in edible fennel tissues, potentially mitigating human health risks.

The limited translocation to edible parts (bulbs) of the evaluated compounds (carbamazepine, climbazole, flecainide and their derivatives) represents a crucial factor to mitigate the potential health risks related to these PhACs.

## 5 Conclusion

This study provides the first analysis of the behavior of selected pharmaceutical compounds in fennel crop irrigated with water containing PhACs. The results highlight that: i) all three PhACs accumulated in soil at high irrigation concentrations, with climbazole and flecainide showing greater persistence even at low doses; ii) carbamazepine was more readily absorbed by roots, exhibiting BCF values >1, yet its movement to aerial plant parts remained limited; iii) climbazole and flecainide showed low bioaccumulation (BCF<1) and negligible translocation to edible tissues (TF≈0), remaining largely confined to soil or roots; iv) the low translocation rates of all tested PhACs suggest the possible presence of “physiological barriers” that limit their movement to edible fennel parts, potentially lowering the risk of human exposure via consumption; v) regarding PhACs metabolites, no compounds attributable to the transformation products of flecainide and climbazole were detected; however, the detection of acridine in the soil, even at low concentrations and as the sole metabolite of carbamazepine, raises concerns regarding its potential long-term entry into the food chain, suggesting the need for frequent evaluation of that compound in the soil-plant system.

The limited translocation of the investigated PhACs to the edible bulb suggests that fennel may represent a low-risk crop for treated wastewater reuse. These findings support the implementation of crop-specific risk assessments within current wastewater reuse regulations (e.g., EU Regulation 2020/741), emphasizing that not all crops pose the same level of risk for human exposure. This aspect is particularly important given that, in many European countries, integrated systems for managing non-conventional water sources, including treated wastewater irrigation, are currently under evaluation.

Future research should extend this evaluation across multiple crop cycles, various environmental conditions, and additional pharmaceutical compounds to better understand the fate of PhACs, and their metabolites, in soil-plant system.

## Data Availability

The original contributions presented in the study are included in the article/**Supplementary Material**. Further inquiries can be directed to the corresponding author.
