# Supplementary material for: Soil accumulation and plant uptake of pharmaceutical active compounds and related metabolites from irrigation water in fennel (Foeniculum vulgare Mill.)
Source: Front Plant Sci. 2026 Feb 23;17:1664441. doi: 10.3389/fpls.2026.1664441 (PMC12968023; doi:10.3389/fpls.2026.1664441)
Supplement: Supplementary file 1 [file Table1.docx]

Soil Accumulation and Plant Uptake of Pharmaceutical Active Compounds (PhACs) and related metabolites from Irrigation Water in Fennel (*Foeniculum vulgare* Mill.)

Giuseppe Gatta^1^, Francesco De Mastro^2*^, Federica Carucci^3^, Michele Perniola^4^, Michele Denora^4^, Gennaro Brunetti^2^, Anna Gagliardi^1^, Marcella M. Giuliani^1^

^1^ Department of Agricultural Sciences, Food, Natural Resources and Engineering (DAFNE), University of Foggia, Foggia 71122, Italy

^2^ Department of Soil, Plant, and Food Science, University of Bari, Bari 70126, Italy

^3^ Department of Agriculture, Forest, Food and Environmental Sciences, University of Basilicata, Viale dell'Ateneo Lucano 10, 85100 Potenza, Italy.

^4^ Department of European and Mediterranean Cultures, University of Basilicata, Matera 75100, Italy

*** Correspondence:** Francesco De Mastro ([francesco.demastro@uniba.it](mailto:francesco.demastro@uniba.it))

This document provides more detailed information on the main paper mentioned above.

The following information is included:

| **Table** | **Content** |
| --- | --- |
| Table S_1_ | Table S1 - Quantification limits (LOQ), detection (LOD) and absolute recovery percentage in soil and plant. |
| Table S_2_ | Table S2 - The mean content of PhACs (ng g^-1^ dry weight) in the soil, plant (root, leaf and bulb) of fennel crop according to different irrigation water content |
| Table S_3_ | Table S3 - Translocation factor (TF) values of the examined pharmaceuticals (PhACs) in different plant parts (root, leaf and marketable yield), associated with irrigation treatments (Control_0.0_, Low_0.5_, Low_2.0_, Low_200_ and Low_600_). The data for each PhACs are shown as the mean ± standard error, based on three replications. |

Table S1 - Quantification limits (LOQ), detection (LOD) and absolute recovery percentage in soil and plant.

| **Compounds** | **Soil** | **Plant** | **Soil** | **Plant** | **Soil** | **Plant** |
| --- | --- | --- | --- | --- | --- | --- |
|  | **LOQ**  (ng g^-1^) | | **LOD**  (ng g^-1^) | | **Recovery %** | |
| **Carbamazepine** | 0.2 | 0.6 | 0.1 | 0.2 | 95 | 81-92 |
| Acridine | 0.2 | 0.6 | 0.1 | 0.2 | 79 | 80-95 |
| 3-Hydroxycarbamazepine | 0.3 | 0.5 | 0.1 | 0.2 | 95 | 78-90 |
| 10,11-dihydro-10-hydroxycarbamazepine | 0.2 | 0.4 | 0.1 | 0.1 | 85 | 85-101 |
| Carbamazepine  10,11-epoxide | 0.3 | 0.5 | 0.1 | 0.2 | 82 | 77-95 |
| 10,11-dihydro-10,11-dihydroxy carbamazepine | 0.2 | 0.6 | 0.1 | 0.2 | 79 | 78-103 |
| **Climbazole** | 0.4 | 0.7 | 0.1 | 0.2 | 98 ± 9 | 89-109 |
| OH-Climbazole | 0.4 | 0.6 | 0.1 | 0.2 | 108 ± 5 | 79-89 |
| **Flecainide** | 0.5 | 0.6 | 0.2 | 0.2 | 89 ± 3 | 82-98 |

Table S2 - The mean content of PhACs (ng g^-1^ dry weight) in the soil, plant (root, leaf and bulb) of fennel crop according to different irrigation water content.

| **PhACs** | **PhACs water content**^††^ | **Matrix** | | | |
| --- | --- | --- | --- | --- | --- |
|  |  | Soil | Plant | | |
|  |  |  | Root | Leaf | Bulb |
|  | Control_0.0_ | <LOQ | <LOQ | <LOQ | <LOQ |
|  | Low_0.5_ | <LOQ | <LOQ | <LOQ | <LOQ |
| Carbamazepine | Low_2.0_ | <LOQ | <LOQ | <LOQ | <LOQ |
|  | High_200_ | 42.9±2.3 | 208.9±7.4 | <LOQ | 13.8±0.82 |
|  | High_600_ | 173.5±6.0 | 458.7±10.5 | <LOQ | 36.1±0.56 |
|  | Control_0.0_ | 0.0±0.0 | 0.0±0.0 | 0.0±0.0 | 0.0±0.0 |
|  | Low_0.5_ | <LOQ | <LOQ | <LOQ | <LOQ |
| Acridine^†^ | Low_2.0_ | <LOQ | <LOQ | <LOQ | <LOQ |
|  | High_200_ | 9.1±0.3 | 5.4±0.2 | <LOQ | <LOQ |
|  | High_600_ | 9.3±0.2 | 5.8±0.1 | <LOQ | <LOQ |
|  | Control_0.0_ | 0.0±0.0 | 0.0±0.0 | 0.0±0.0 | 0.0±0.0 |
|  | Low_0.5_ | 2.0±0.2 | <LOQ | <LOQ | <LOQ |
| Climbazole | Low_2.0_ | 3.9±0.6 | <LOQ | <LOQ | <LOQ |
|  | High_200_ | 190.5±5.6 | 23.4±1.4 | <LOQ | <LOQ |
|  | High_600_ | 418.7±4.3 | 41.5±1.2 | 26.75±1.7 | 11.4±1.12 |
|  | Control_0.0_ | 0.0±0.0 | 0.0±0.0 | 0.0±0.0 | 0.0±0.0 |
|  | Low_0.5_ | 5.1±0.5 | <LOQ | <LOQ | <LOQ |
| Flecainide | Low_2.0_ | 5.3±0.7 | <LOQ | <LOQ | <LOQ |
|  | High_200_ | 124.6±7.4 | 55.9±4.7 | <LOQ | <LOQ |
|  | High_600_ | 497.9±15.9 | 240.5±18.4 | <LOQ | <LOQ |

^†^ Metabolite of the carbamazepine

^††^ Low_0.5_: PhAC concentration at 0.5 μg L^-1^; Low_2.0_: PhAC concentration at 2.0 μg L^-1^; High_200_: PhAC concentration at 200 μg L^-1^; High_600_: PhAC concentration at 600 μg L^-1^

LOQ, PhACs content below to the Limit of Quantification

The values reported are the mean value ± standard error (three replications)

Table S3 - Translocation factor (TF) values of the examined pharmaceuticals (PhACs) in different plant parts (root, leaf and marketable yield), associated with irrigation treatments (Control_0.0_, Low_0.5_, Low_2.0_, Low_200_ and Low_600_). The data for each PhACs are shown as the mean ± standard error, based on three replications.

| **Translocation factor (TF) ^†^** | **PhACs** | | | |
| --- | --- | --- | --- | --- |
|  | Carbamazepine | Acridine | Climbazole | Flecainide |
| TF _(leaf/root)_ |  |  |  |  |
| Control_0.0_ | 0.0±0.0 | 0.0±0.0 | 0.0±0.0 | 0.0±0.0 |
| Low_0.5_ | 0.0±0.0 | 0.0±0.0 | 0.0±0.0 | 0.0±0.0 |
| Low_2.0_ | 0.0±0.0 | 0.0±0.0 | 0.0±0.0 | 0.0±0.0 |
| High_200_ | 0.0±0.0 | 0.0±0.0 | 0.0±0.0 | 0.0±0.0 |
| High_600_ | 0.0±0.0 | 0.0±0.0 | 0.64±0.01 | 0.0±0.0 |
| TF _(bulb/root)_ |  |  |  |  |
| Control_0.0_ | 0.0±0.0 | 0.0±0.0 | 0.0±0.0 | 0.0±0.0 |
| Low_0.5_ | 0.0±0.0 | 0.0±0.0 | 0.0±0.0 | 0.0±0.0 |
| Low_2.0_ | 0.0±0.0 | 0.0±0.0 | 0.0±0.0 | 0.0±0.0 |
| High_200_ | 0.06±0.003 | 0.0±0.0 | 0.0±0.0 | 0.0±0.0 |
| High_600_ | 0.08±0.001 | 0.0±0.0 | 0.27±0.01 | 0.0±0.0 |

^†^ Control_0.0_, control by Irrigation with freshwater; Low_0.5_, freshwater spiked with PhAC at a concentration of 0.5 µg L^−1^; Low_2.0,_ freshwater spiked with PhACs at a concentration of 2 µg L^−1^; High_200_, freshwater spiked with EC at a concentration of 200 µg L^−1^; High_600_, freshwater spiked with PhACs at a concentration of 600 µg L^−1^.
